# Arteries Stiffen With Age, but Can Retain an Ability to Become More Elastic With Applied External Cuff Pressure

**DOI:** 10.1097/MD.0000000000001831

**Published:** 2015-10-16

**Authors:** Chengyu Liu, Dingchang Zheng, Alan Murray

**Affiliations:** From the Institute of Cellular Medicine, Newcastle University, Newcastle upon Tyne, NE1 4LP, UK (CL, DZ, AM); School of Control Science and Engineering, Shandong University, Jinan, 250061, China (CL); and Health and Well Being Academy, Faculty of Medical Science, Anglia Ruskin University, Chelmsford, CM1 1SQ, UK (DZ).

## Abstract

It is accepted that arterial compliance decreases with age, with changes in the arterial pulse shape measured at the periphery. The aim of this study was to determine the relationship between arterial transmural pressure changes and changes in peripheral finger pulse shape characteristics for both older and younger subjects.

Finger photoplethysmographic pulses were recorded noninvasively from the right index fingers of 100 healthy normotensive subjects. Their median age was 43 years (range 20–71 years) allowing two distinct age groups to be compared (older group ≥45 and younger group < 45 years). Arterial transmural pressures on the whole right arm were reduced with a 50 cm long cuff inflated to 10, 20, 30, and 40 mmHg. Pulse maximum amplitude and rise time were calculated for each age group, and for each cuff pressure level.

Gradual and significant decreases in both pulse maximum amplitude and rise time were found with increasing cuff pressure for both age groups. With an external cuff pressure of 40 mmHg, there was an average maximum amplitude and rise time decrease of 27.1% (*P* < 0.001) and 7.5% (*P* < 0.001) respectively. The changes in the older group were significantly greater than those in the younger group for maximum amplitude (30.3% vs 24.4%, *P* = 0.006), but not for rise time (8.0% vs 6.7%, *P* = 0.23).

Our results show that arterial compliance of the arm artery increases with reduced transmural pressure for both older and younger groups, and demonstrate that the aged arm artery can become more elastic with applied external cuff pressure.

## INTRODUCTION

The arterial pulse can be measured by various techniques. Photoplethysmography (PPG) has been widely used due to its simple operation, inexpensive cost, and ability to provide valuable information about the peripheral circulation.^1,2^ It is accepted that cardiovascular mortality is strongly linked to arterial properties.^3^ Pulse shape characteristics have been accepted as risk indicators of cardiovascular diseases.^4,5^

The ability to quantify the arterial pulse shape characteristics and thereby explore the influence of factors on the pulse shape is important. It is accepted that age plays an important role on arterial properties.^6^ There are many published research studies concerning the age effect on pulse transit time (PTT) and pulse wave velocity (PWV). PTT decreases with age whereas PWV increases.^7,8^ The age-related changes in pulse shape characteristics have also been reported in the relatively large compliant arteries, such as carotid arteries.^9^ However, for the limb arteries, the effect of age on pulse shape characteristics is still clinically controversial. It has been shown that pulse shape characteristics change in the proximal brachial artery^10,11^ and at the finger PPG^12–14^ with ageing, while others reported no age effect for the pulse shape in the distal brachial artery.^15^

Zheng and Murray^16^ have demonstrated that the changes of PTT and PWV in peripheral arteries could be doubled with the use of a lower transmural pressure induced by a specialized long cuff on the whole arm. Previous studies also showed that the elastic properties of the brachial artery change with different transmural pressures induced by raising the arm or by an external cuff on the forearm.^17–19^ However, to the best of our knowledge, there have been no similar studies specifically designed to examine the effect of different transmural pressures on pulse shape change for peripheral arteries from different age groups.

**FIGURE 1:**
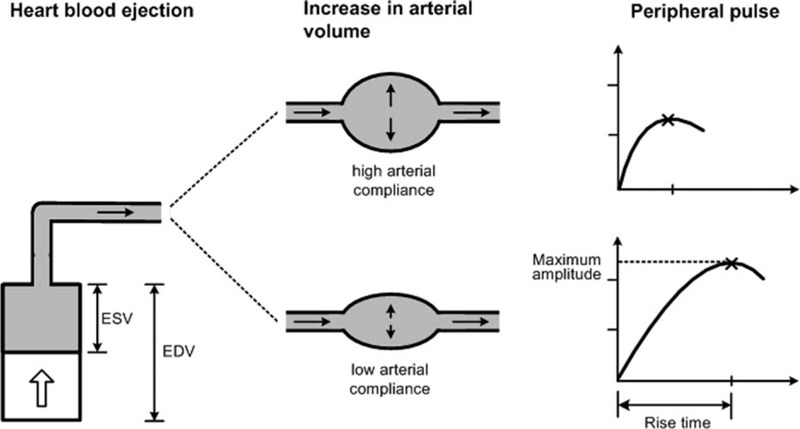
Model of hypothesis.

This study therefore aimed to quantify finger PPG shape changes with different transmural pressures induced by a specialized long cuff on the whole arm for both older and younger groups, and to compare the differences in changes between the 2 groups. Our hypothesis is illustrated in Figure 1. As the heart beats, the ventricular volume reduces from end diastolic volume. At the end of systole, the heart has completed blood ejection and has its minimum volume (end systolic volume). If the peripheral arterial system has relatively high compliance, a larger proportion of the ejected blood will expand the peripheral arteries and hence the peripheral pulse delivered to the end of the peripheral artery system would be expected to have lower maximum amplitude. In addition, the rise time would be expected to be short as the ejected blood would be taken up by the high compliance relatively quickly. So, we aimed to detect the waveform change of peripheral pulse to reflect the arterial compliance.

## METHODS

### Subjects

In total, 100 healthy normotensive volunteers with no history of cardiovascular disease were studied. They were mainly from staff, students, and their relatives of Newcastle Hospitals and Newcastle University. All the subjects were divided into 2 age groups: older group (age ≥ 45 years, 46 subjects) and younger group (age < 45 years, 54 subjects). Subject demographic information including age, gender, height, body mass, body mass index, and blood pressures are summarized in Table 1. Their means and standard deviations (SDs) for each group are also given. The investigation conformed to the principles in the Declaration of Helsinki (World Medical Association 2000). The study received ethical permission, and all subjects gave their written informed consent.

**TABLE 1:**
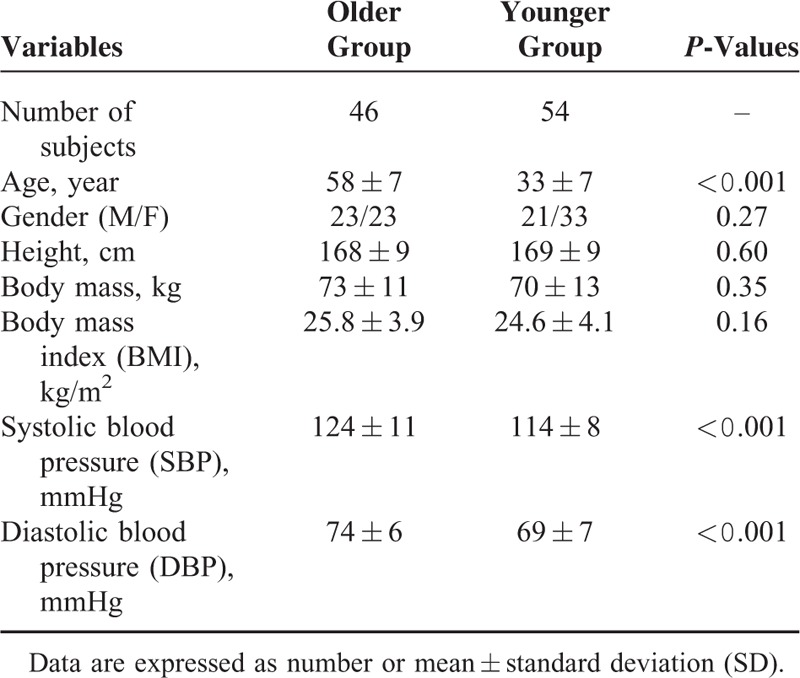
Selected Variables of All Subjects, the *P*-Values Measure the Separation Between Older (≥45 years) and Younger (<45 years) Groups

### Experimental Procedure

**FIGURE 2:**
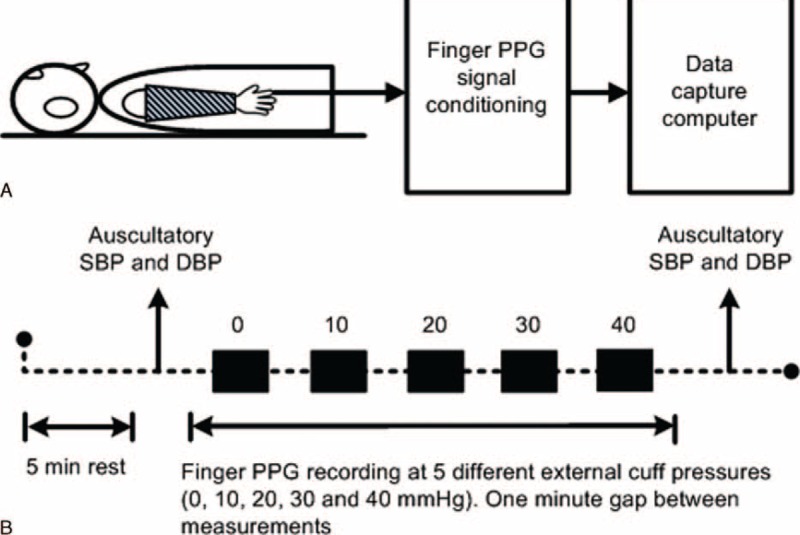
Schematic diagram and experimental procedure for the measurement system with the long cuff. (A) Schematic diagram, (B) experimental procedure.

Figure 2 gives a schematic diagram illustrating the measurement system and experimental procedure. To apply different external pressures along the whole arm artery and to generate different transmural pressures across the arterial wall, we used a specially designed cuff (50 cm long), manufactured by A C Cossor & Son (Surgical) Ltd. Its wrist end circumference was 17 cm, and the circumference of the top end for the upper arm was 31 cm.^16^ In order to indicate the applied external cuff pressure, the Accoson Greenlight 300 sphygmomanometer was used, which can display cuff pressure from 0 to 300 mmHg.

All the measurements were taken in a temperature-controlled measurement room, which provided a stable temperature of 23 ± 1 °C. Before the formal recording, each subject lay supine on a measurement couch for a 5 minutes rest period to allow cardiovascular stabilization. PPG probes were attached to the right index fingertip, and the specially designed long cuff was then wrapped around the whole right arm. All the measurements were performed by an experienced operator to ensure the probes and the long cuff were attached with similar tightness for all subjects and to ensure the fingers were not cold. During the measurement, the arms were kept parallel to the body and as still as possible, with regular and gentle breathing.

**FIGURE 3:**
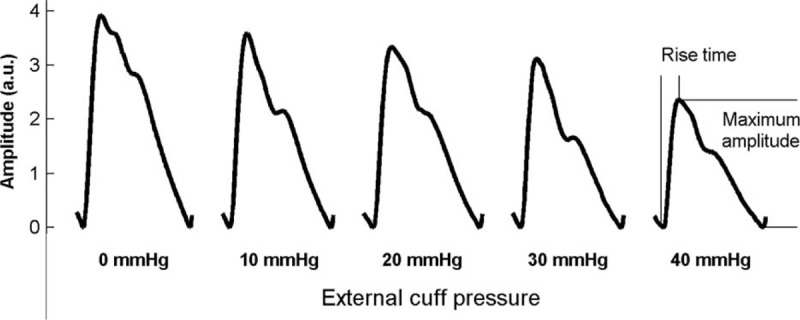
Examples of typical photoplethysmography (PPG) pulses extracted from a subject at 5 different external cuff pressures. Two pulse shape characteristics, maximum amplitude and rise time, were extracted for each subject and each external cuff pressure.

Five separate recordings were performed, each with a different external cuff pressure (0, 10, 20, 30, and 40 mmHg) applied to the whole right arm through the long cuff, with a 1 minute gap for rest between each recording. For each recording, the finger PPG was recorded at a sample rate of 2500 Hz for 120 seconds. Figure 3 gives an example of typical finger PPG waveforms with the 5 external cuff pressures from 1 subject.

Auscultatory systolic and diastolic blood pressures (SBP and DBP) were recorded manually at the beginning and end of the measurement from the right upper arm. The maximum resting SBP and DBP from all the subjects were less than 140 and 90 mmHg, respectively. The overall mean and SD values of SBP and DBP are given in Table 1.

### Data and Statistical Analysis

For each subject at each cuff pressure recording, the pulse feet and pulse peaks were identified in each beat. Any ectopic beats were excluded using a method described.^20^ As shown in Figure 3, for each beat pulse, 2 indices were defined: the maximum amplitude and the rise time. The maximum amplitude is the amplitude different between the pulse foot and pulse peak. The rise time is the time interval between the pulse peak and pulse foot. For statistical analysis, the overall means and standard errors of the indices for each age group were obtained. For each age group, these 2 indices were compared between 0 mmHg and the 4 higher (10, 20, 30, and 40 mmHg) cuff pressures, and all statistical analysis was performed on paired data. A value of *P* < 0.05 was considered statistically significant.

To form the group mean pulse shape, for each subject at each cuff pressure recording, each beat pulse was normalized to a pulse width of 1000 sample points using cubic spline interpolation, and then averaged across all beats within each subject to obtain the individual average for all beat pulses. The average for these individual width normalized pulses in each group formed the group mean pulse shape.

## RESULTS

After normalization for each beat using cubic spline interpolation, the mean pulse shapes for both older and younger groups were obtained. At 0 mmHg cuff pressure the mean pulse shape from older group was much higher in amplitude and wider in shape in comparison with the younger group. However, with 40 mmHg external pressure, the mean pulse shape from the older group shrank to a similar shape as the younger group. To further understand the mechanism, the maximum amplitude and the pulse rise time were analyzed.

**FIGURE 4:**
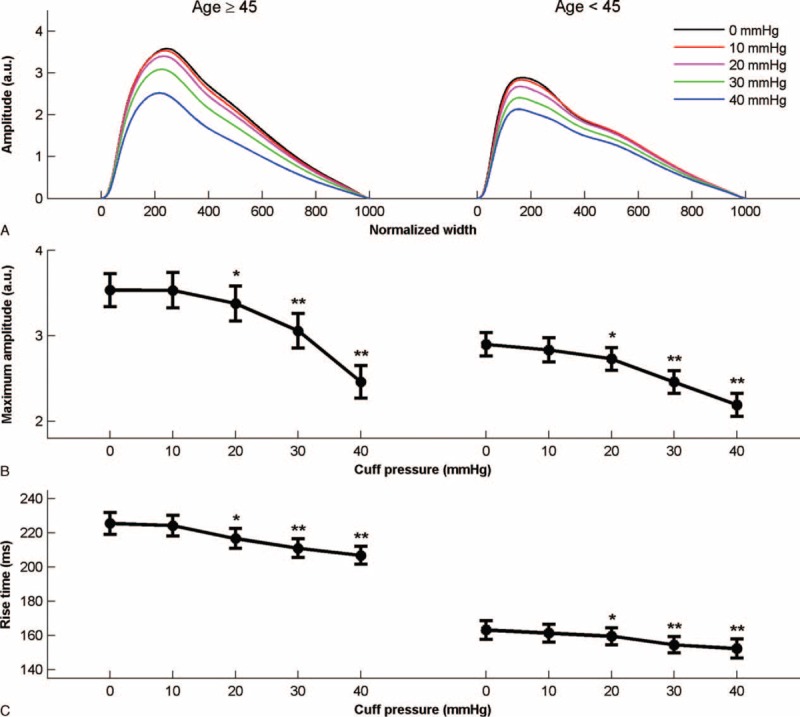
Group mean photoplethysmography (PPG) pulse shape characteristics for the older and younger subjects at 5 external cuff pressures. (A) PPG pulse shape, (B) overall maximum amplitude characteristics, and (C) overall rise time characteristics. Compared with 0 mmHg cuff pressure in each group, statistically significant differences at the *P* < 0.05 level are marked as “∗” and *P* < 0.01 level are marked as “∗∗.”.

Figure 4A shows the mean finger pulse shape for both older and younger groups at the 5 cuff pressures with normalized width. The pulses described the overall shape characteristics for each group at each of the 5 cuff pressures to allow the pressure-related shape changes to be observed and compared.

Figure 4B shows the means and standard errors of overall pulse maximum amplitude for each cuff pressure. Table 2 gives the change to the values relative to 0 mmHg cuff pressure. The pulse maximum amplitude decreased with increasing cuff pressure for both older and younger groups. When compared with the measurement from 0 mmHg cuff pressure in each group, statistically significant decreases were observed at 20 (*P* = 0.02), 30 (*P* < 0.001), and 40 (*P* < 0.001) mmHg cuff pressures. For the older group, the ratios of pulse maximum amplitude between the 4 higher cuff pressures (10, 20, 30, and 40 mmHg) and 0 mmHg cuff pressure were 99.7%, 95.8%, 86.7%, and 69.7%, respectively. For the younger group, they were 97.6%, 94.2%, 84.8%, and 75.6%, respectively. In addition, the pulse maximum amplitudes in the older group were higher than those in the younger group at all 5 cuff pressures. The pulse maximum amplitude at 40 mmHg cuff pressure in the older group was lower than that at 0 mmHg cuff pressure in the younger group (2.5 ± 0.19 vs 2.9 ± 0.14 a.u., *P* < 0.001).

**TABLE 2:**
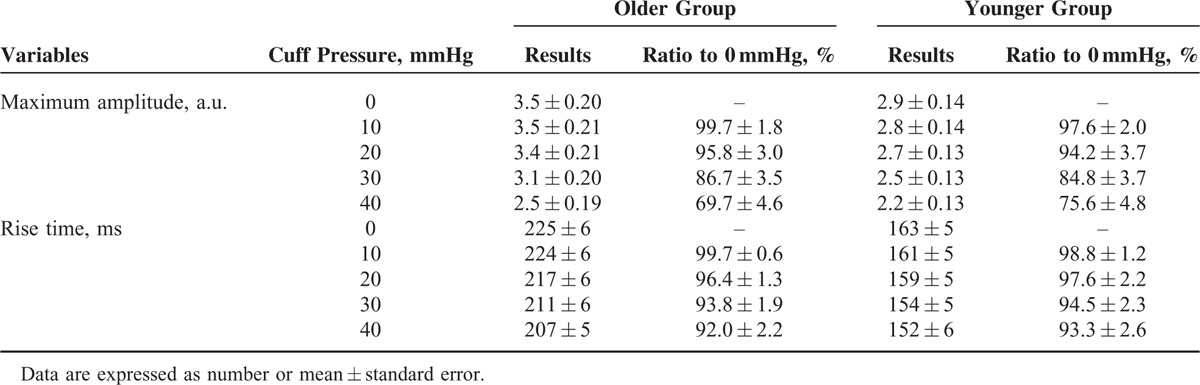
Results of Maximum Amplitude and Rise Time from 5 Cuff Pressures for Both Older and Younger Groups

Figure 4C shows the means and standard errors of overall rise time for each cuff pressure level. Table 2 also gives its relative change to the values at 0 mmHg cuff pressure. The rise time also decreased with the increase of cuff pressure for each group. When compared with the measurement from 0 mmHg cuff pressure in each group, statistically significant decreases were also observed at 20 (*P* = 0.01), 30 (*P* < 0.001), and 40 (*P* < 0.001) mmHg cuff pressures. For the older group, the ratios of rise time between the 4 higher cuff pressures (10, 20, 30, and 40 mmHg) and the 0 mmHg cuff pressure were 99.7%, 96.4%, 93.8%, and 92.0%, respectively. For younger group, they were 98.8%, 97.6%, 94.5%, and 93.3%, respectively. The changes of rise time were smaller than those of pulse maximum amplitude. For the comparison of 2 groups, the older group always has a longer rise time than the younger group. However, the external 40 mmHg cuff pressure did not make the rise time in the older group lower than the rise time with 0 mmHg cuff pressure in the younger group.

With the external cuff pressure increasing from 0 to 40 mmHg, the transmural pressure is reduced by the same amount, and it can be seen from Table 2, that the maximum amplitude and rise time of the peripheral optical pulse decreased on average by 27.1% (*P* < 0.001) and 7.5% (*P* < 0.001), respectively. The changes in the older group were more significant than those in the younger group for maximum amplitude (30.3% vs 24.4%, *P* = 0.006) but not for rise time (8.0% vs 6.7%, *P* = 0.23).

## DISCUSSION

This study showed that, with increasing external cuff pressure applied on the whole arm, the maximum amplitude and rise time of the peripheral optical pulse measured from the fingertip decreased gradually in both older and younger groups. The specially designed long air-filled pressure cuff makes it possible to investigate the pulse shape characteristic changes over a range of low transmural pressures.

The desire for simple, economical, convenient, and noninvasive cardiovascular assessment techniques has been a stimulus to reinvestigate the peripheral PPG pulses.^2^ Our current study expanded the physiological investigation for a better understanding of the optical pulse shape characteristic changes with different mechanical conditions across the arterial wall, with a particular focus on the effect of different transmural pressures and its interaction with aging.

The optical pulse shape characteristics reflect the change of blood volume, arterial compliance, peripheral resistance, and physiological condition caused by the change in the properties of an arteriole.^13,16,18,21,22^ It is also influenced by sympathetic activity as well as temperature variations.^23^ It has been reported that pulse amplitude is directly proportional to local vascular distensibility over a remarkably wide range of cardiac outputs.^24^ It has also been suggested that pulse amplitude is potentially a more suitable measure than pulse arrival time for estimating continuous blood pressure.^25,26^ The pulse amplitude is an indicator of the pulsatile changes in blood volume caused by arterial blood flow around the measurement site.^27^

We hypothesize that, if the peripheral arterial system had relatively higher compliance, a larger proportion of the ejected blood would expand the peripheral arteries and hence the finger PPG pulse would be expected to have lower maximum amplitude. In the present study, it has been observed in both older and younger groups that the pulse maximum amplitude decreased with increasing external cuff pressure, indicating increased compliance with applied external cuff pressure. The decrease of maximum amplitude in the finger pulse with the increase of external finger forces has also been reported.^28^ However, these forces were directly applied to the fingertips, not to the whole arm as in this study. It is also worth noting that, for older subjects, the maximum amplitude at the high external pressure of 40 mmHg could reach that of the younger subjects at normal atmospheric pressure, demonstrating that the aged arteries have the ability to become more elastic with applied external cuff pressure.

We also hypothesize that, for peripheral arteries with high compliance, the rise time of the measured pulse would be expected to be shorter as the ejected blood would be taken up relatively quickly. We observed the decrease of the pulse rise time for both older and younger groups with external pressure, providing further confirmation of the increase of arterial compliance with applied external pressure. However, with 40 mmHg external cuff pressure applied for the older subjects, the measured pulse rise time did not reach the level at normal atmospheric pressure in younger subjects, suggesting that the pulse rise time was not only related to arterial compliance, but also to peripheral resistance, as the resistance of arterioles may change little with different external cuff pressures.

Our study confirms the expectations of our hypothesis, with the external cuff pressure increasing, peripheral arterial compliance increases, resulting in blood volume pulsations decreasing to a relatively low pulse amplitude and a low pulse rise time. The applied external cuff pressure could be a potential method for investigating arterial compliance in clinical application.
